# Supplementary material for: Tat-hspb1 Suppresses Clear Cell Renal Cell Carcinoma (ccRCC) Growth via Lysosomal Membrane Permeabilization
Source: Cancers (Basel). 2022 Nov 21;14(22):5710. doi: 10.3390/cancers14225710 (PMC9688814; doi:10.3390/cancers14225710)
Supplement: Supplementary file 1 [file cancers-14-05710-s001.zip › Table S1.pdf]

Table S1. Baseline characteristics

	Patient-1	Patient-2	Patient-3
Age	61	60	65
Gender	Female	Male	male
BMI	23	25.4	23.8
Hypertension	No	No	No
Diabetes	No	No	No
Pre-nephrectomy creatinine, mg/dl	0.65	0.90	0.63
Tumor size, cm	2	3	4.7
Histologic subtype	Clear cell	Clear cell	Clear cell
WHO/ISUP grade	1	2	1
Pathologic T stage	T1a	T1a	T1b
Pathologic N status	N0	N0	N0
Pathologic M status	M0	M0	M0
