# Supplementary material for: Tat-hspb1 Suppresses Clear Cell Renal Cell Carcinoma (ccRCC) Growth via Lysosomal Membrane Permeabilization
Source: Cancers (Basel). 2022 Nov 21;14(22):5710. doi: 10.3390/cancers14225710 (PMC9688814; doi:10.3390/cancers14225710)
Supplement: Supplementary file 1 [file cancers-14-05710-s001.zip › Table S2.pdf]

Table S2. Differentially expressed peptides

Peptide Sequence	Gene Name	Molecular Weight	Log2 FC	P value
GDPNYLHRMTTL	PPP2R1A	1416.682	5.67766676	0.00205534
ELGYTVKKHLQDLSGRISR	LRPAP1 A2MRAP	2199.2124	5.90514613	0.04231093
TGIFTDQVLSVLKGEE	APOC2 APC2	1734.904	-4.5965447	0.04992282
KLKLGQSVTTIPTVGF	ARF6	1687.9873	-2.4694816	0.02181921
TATLRPYLSA	ARPC4 ARC20	1091.5975	4.8065138	0.00302846
SRIYHDGALR	ARPIN C15orf38	1186.6207	2.68767481	0.02008305
VPPVQVSPLIKLGRYSALF	ATP5ME ATP5I ATP5K	2083.2194	-2.3655474	0.01049308
VPPVQVSPLIKLGRYSALFL	ATP5ME ATP5I ATP5K	2196.3034	-3.324978	0.04489413
MDDFERRRELR	CALD1 CAD CDM	1521.747	-1.9147675	0.03315676
EGPIPEVLKNYMDAQY	CTSD CPSD	1865.887	5.78643953	0.03086903
GPIPEVLKNYMDAQY	CTSD CPSD	1736.8444	-2.178019	0.00424939
KLVDQNIFS	CTSD CPSD	1062.571	2.18342725	0.03617949
VERQVFGEATKQPGIT	CTSD CPSD	1758.9264	-2.1766193	0.0351754
VSSFLPWIR	CTSG	1103.6128	-8.0575442	0.01002414
HSYSQMLLFPY	CPA3	1384.6486	-7.0614172	0.01319404
QDLQQKVVY	CD68	1119.5924	3.45996631	0.00712208
AGQAFRKFLPLFDRVLVERSA	HSPE1	2419.3488	-2.8000586	0.02304149
AGQAFRKFLPLFDRVLVERSAAETV	HSPE1	2819.5446	-7.8584308	0.02962995
SNMEKHLFNLK	CHMP1B C18orf2	1359.6969	-1.5392297	0.00087304
PITVTVPVEVSRKN	CLU APOJ CLI KUB1 AAG4	1537.8828	-5.835052	0.00075405
IEWNLSPVGRVTPKEWRNQ	COX7B	2308.2076	-5.7429767	0.01967085
VGDEDFVH	CSTB CST6 STFB	916.39266	7.3376869	0.00373521
AAAAKPNNLSLVVHGPGDLRLENY	SORD	2518.3292	-3.6397948	0.0014406
SSTLAKIAEIEAEMARTQKN	DRG1 NEDD3	2190.1314	5.28662178	0.01190173
TTEVGSVSEVKKDS	EPB41L2	1464.7308	-5.9088604	0.00247452
AEYTRLHNALALIRLR	EHHADH ECHD	1909.101	-2.8634944	0.02945852
VNFTVDQIR	EEF2 EF2	1090.5771	2.44922399	0.03321227
SGPGLSPGMVR	FLNA FLN FLN1	1056.5386	-7.815948	0.03878548
SSSHSRAGQSAAGAAP	FLNA FLN FLN1	1440.6706	2.54973275	0.03092987
NDHFVKLISWY	GAPDH GAPD CDABP0047 OK/SW-cl.12	1420.7139	-7.9989607	0.03983395
PYLLISTQIR	GCHFR GFRP	1202.7023	-6.3622352	0.03697474
DTAKKPIIGILMQ	GGH	1426.8218	-8.0983236	0.02149374
DTAKKPIIGILMQKCRNK	GGH	2056.1649	-8.0349904	0.00393996

TMSLLAANNLLAGLRGEPMPSELKL	GRHPR GLXR MSTP035	2638.4186	-3.8058907	0.02491768
AFLASPEYVNLPINGNGKQ	GSTP1 FAEES3 GST3	2031.0425	3.73907954	0.02229884
LKAFLASPEYVNLPINGNGKQ	GSTP1 FAEES3 GST3	2272.2216	4.77936577	0.00114134
VGENADSQIKLSIK	H1-0 H1F0 H1FV	1500.8148	-4.2215842	0.00480644
SETAPAAPAAPAPAEKTPVKKK	H1-4 H1F4 HIST1H1E	2159.195	-1.6332517	0.01960791
SETAPAAPAAPAPAEKTPVKKKARK	H1-4 H1F4 HIST1H1E	2514.4282	-1.9828011	0.046213
DALTNAVAHVDDMPN	HBA1; HBA2	1581.7093	5.05535494	0.02045441
VLSPADKTNVKAAWGKVGA	HBA1; HBA2	1911.0578	-2.2654815	0.04670935
MLGMIKNS	HEBP1 HBP	892.45104	-6.2760283	0.00089471
PSSGLGVTKQDLGPVPM	CD74 DHLAG	1681.8709	2.04218712	0.00973222
PAVAAPAYSRALSRQL	HSPB1 HSP27 HSP28	1669.9264	-1.1027559	0.03454815
RGPSWDPFRDWYPHSRLFDQAFGL	HSPB1 HSP27 HSP28	2949.4099	-3.8175667	0.0211277
QIIQEKRGY	IFITM1 CD225 IFI17	1133.6193	-2.7587731	0.03642006
GPASRSVETL	PKM OIP3 PK2 PK3 PKM2	1015.5298	2.11158893	0.04202609
VADTPENLRLK	LASP1 MLN50	1254.6932	7.48544477	0.02326741
SHSHMAETASPTPLN	MS4A4A 4SPAN1 CD20L1 MS4A4 HDCME31P	1578.7097	3.12337061	0.03381769
GPIITVHPKEIRTF	MAN2B2 KIAA0935	1606.9195	-7.423776	0.00031428
AAAAELSLLEKSLGLSKGNK	EEF1E1 AIMP3 P18	1999.1314	-3.2090678	0.00775628
AGVLKKTTGLVG	NDUFA5	1142.7023	-10.861695	0.00513615
ASATRLIQRLRNWASGHDLQGKLQL	NDUFA7	2831.5631	-2.1522029	0.00546259
AGQPGHMPHGGSSN	NEK6	1332.5629	1.59362839	0.02856801
MLSYGERLGSPAVSPLPVRGGHVMR	SLC34A1 NPT2 SLC17A2	2665.3945	-7.4244951	0.04737769
MFPAAPSPRTPGTGSR	NUP133	1628.8093	1.63218961	0.01271464
AAAAAAAAAGAAGGRGSGPGRR	PABPN1 PAB2 PABP2	1864.9728	-6.4569751	0.00227268
ENVPEEEGKVRSTPM	PDZK1IP1 MAP17	1700.8039	-3.9363311	0.01152846
AAVDELGKVLTPTQV	PEBP1 PBP PEBP	1539.8508	-2.4974218	0.02937675
SLSNKLTLDKLDVKGKRVV	PGK1 PGKA MIG10 OK/SW-cl.110	2112.263	1.01117463	0.00056044
ASVAVDPQPSVVTR	PLIN2 ADFP	1424.7623	3.9445329	0.03080169
ASVAVDPQPSVVTRVV	PLIN2 ADFP	1622.8992	7.10804265	0.03185568

ASVAVDPQPSVVTRVVNLP	PLIN2 ADFP	1947.0789	4.16243365	0.04602815
GVENALTKSEL	PLIN2 ADFP	1159.6085	2.97524038	0.02938783
GVENALTKSELL	PLIN2 ADFP	1272.6925	8.006299	0.02557071
GVENALTKSELLVE	PLIN2 ADFP	1500.8035	2.49805975	0.00757893
PQPSVVTRVVNLP	PLIN2 ADFP	1404.8089	5.97580954	0.04915835
SGVENALTKSEL	PLIN2 ADFP	1246.6405	8.72210277	0.04467131
SGVENALTKSELLVE	PLIN2 ADFP	1587.8356	2.70321598	0.04172686
VSSGVENALTKSELLVEQ	PLIN2 ADFP	1901.9946	2.30435023	0.04175864
TDYISKVKE	ARL6IP5 DERP11 JWA PRA2 PRAF3 HSPC127	1081.5655	8.18124387	0.00409668
AGKQAVSASGKWLDGI	UQCRB UQBP	1586.8417	-6.0438036	0.0455667
SRGPGAPASPSASHPQGLDTTPKPH	ARHGAP4 KIAA0131 RGC1 RHOGAP4	2449.2098	5.59087481	0.02189178
IIEPSLRQL	UBA52 UBCEP2	1067.6339	-6.2439444	0.02182805
SVFALTNGIYPHKL	RPL6 TXREB1	1558.8508	2.34932796	0.03256204
NDFGNYNQQPSNYGPMK	HNRNPA2B1 HNRPA2B1	1972.8374	-5.8499087	0.04761849
YGGGNYGPGGSGGSGGYGGRSR	HNRNPA2B1 HNRPA2B1	1975.8521	-5.9237919	0.00580977
MEVKPPPGRPQPDSGR	HNRNPA3 HNRPA3	1746.8835	-6.8860868	0.01196117
NVKGPVREGDVLTLLESEREARRLR	RPS28	2891.6053	6.52259505	0.00255902
ATVTATTKVPEIRDVTR	RUVBL2 INO80J TIP48 TIP49B CGI-46	1857.032	-1.0857608	0.04479179
SLYPSLEDLKVDKVIQAQT	SDCBP MDA9 SYCL	2146.1522	4.88771696	0.03030777
AASAARGAAALRRSINQPVA	SLIRP C14orf156 DC23 DC50 PD04872	1950.0871	-1.8124885	0.02294397
AASAARGAAALRRSINQPVAF	SLIRP C14orf156 DC23 DC50 PD04872	2097.1556	-2.9885889	0.00634614
SINQPVAFVR	SLIRP C14orf156 DC23 DC50 PD04872	1129.6244	-8.3116573	0.02991139
AGGPPKALPSTGPHSLR	SNX30	1641.8951	-5.6058486	0.00277269
AAVPELLQQQEEDRSKLR	SNX5	2109.1178	-2.1019558	0.04102036
KGDGPVQGIINF	SOD1	1243.6561	-2.9583578	0.00231553

AANSSGQGFQNKNR	INIP C9orf80 SSBIP1 HSPC043 HSPC291	1477.7022	2.53871574	0.03065232
ALTSDLGKQIKLK	SULT1C2 SULT1C1	1413.8555	-6.8406154	0.04104699
DEVSRIVGSVE	STAT1	1188.5986	5.71255917	0.01742645
GSNRGASQAGMTGYGRPRQ	TAGLN SM22 WS3-10	1949.9238	-2.4765185	0.04505212
MGSNRGASQAGMTGYGRPRQIIS	TAGLN SM22 WS3-10	2394.1645	-10.075668	0.01865423
VIGLQMGSNRGASQAGMTGYGRPRQ	TAGLN SM22 WS3-10	2591.2809	-3.0168577	0.02161172
AAVKTLNPKAEVARAQA	CCT6A CCT6 CCTZ	1736.9897	-7.6446528	0.00117663
AAVKTLNPKAEVARAQAA	CCT6A CCT6 CCTZ	1808.0268	-1.5252089	0.00766892
MQPASAKWYDRRDYVF	PTGES3 P23 TEBP	2031.9625	6.42437545	0.02712346
SVAGVHTVPGSPQARHR	TNS1 TNS	1754.9288	3.53741073	0.02773984
AGLALEIRSL	TMEM205 UNQ501/PRO1018	1041.6182	-2.1155024	0.04043653
ASASGAMAKHEQILV	VAPA VAP33	1511.7766	-6.379443	0.00605744
ALSDADVQKQIK	ATP6V1E1 ATP6E ATP6E2	1314.7143	-2.0802787	0.02827731
GHKLGLGLEFQA	VDAC1 VDAC	1268.6877	-6.4842381	0.03475725
GGPGTASRP	VIM	798.39842	4.9100206	0.00282526
GGPGTASRPSS	VIM	972.46247	2.00400427	0.04507893
TVETRDGQVIN	VIM	1230.6204	5.28318367	0.0390112
ATTAAPAGGARN	YIPF3 C6orf109 KLIP1	1056.5312	1.99850275	0.04933627
