# Supplementary figures and images for: Tat-hspb1 Suppresses Clear Cell Renal Cell Carcinoma (ccRCC) Growth via Lysosomal Membrane Permeabilization
Source: Cancers (Basel). 2022 Nov 21;14(22):5710. doi: 10.3390/cancers14225710 (PMC9688814; doi:10.3390/cancers14225710)

Figure. S1 The effect of Tat-hspb1 and Sunitinib

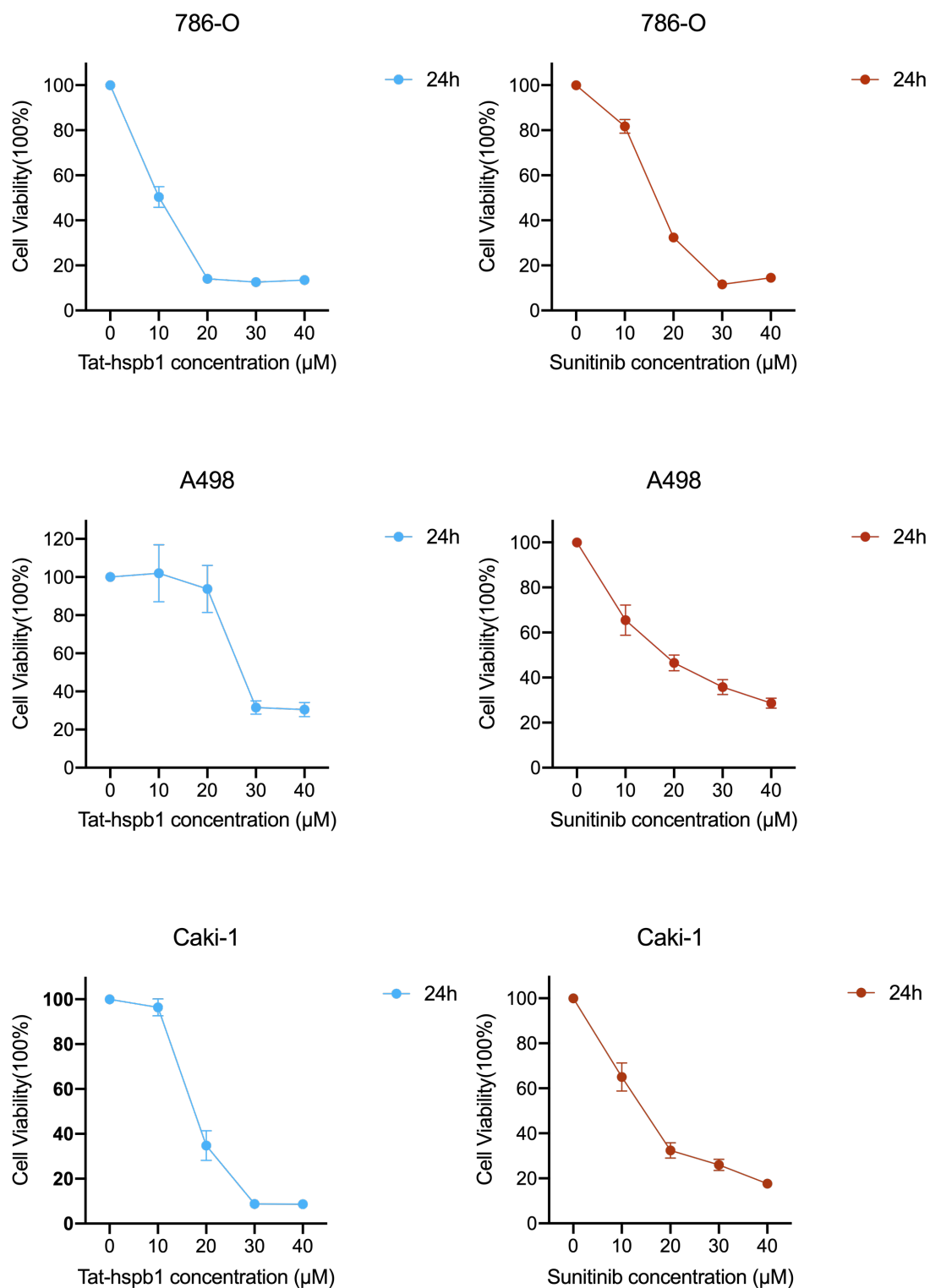

Supplement: Supplementary file 1 [file cancers-14-05710-s001.zip › Fig. S1.pdf]

Whole image of WB in Fig.4C

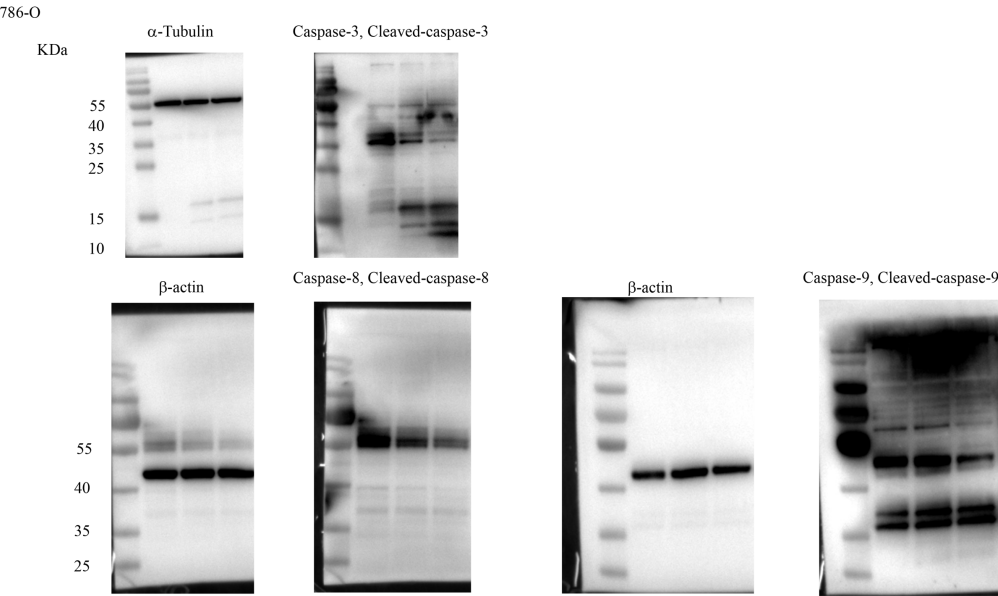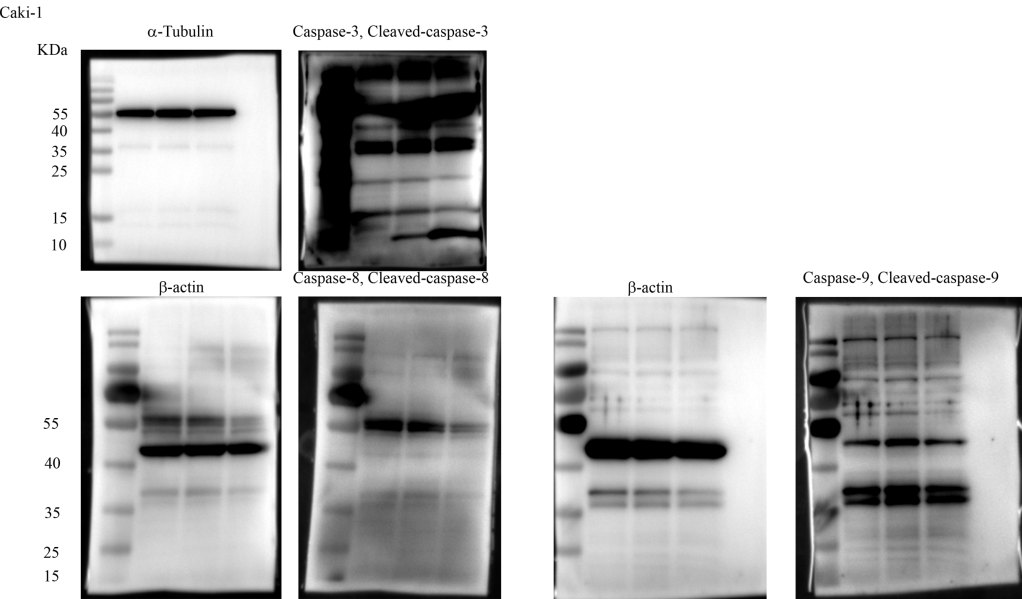

Supplement: Supplementary file 1 [file cancers-14-05710-s001.zip › Fig. S2.pdf]
